# The Protective Effects of Nutraceutical Components in Methotrexate-Induced Toxicity Models—An Overview

**DOI:** 10.3390/microorganisms10102053

**Published:** 2022-10-18

**Authors:** Gheorghe-Eduard Marin, Maria-Adriana Neag, Codrin-Constantin Burlacu, Anca-Dana Buzoianu

**Affiliations:** 1 Faculty of Medicine, Iuliu Hatieganu University of Medicine and Pharmacy, 400349 Cluj-Napoca, Romania; 2 Department of Pharmacology, Toxicology and Clinical Pharmacology, Iuliu Hatieganu University of Medicine and Pharmacy, 400337 Cluj-Napoca, Romania

**Keywords:** methotrexate, gut microbiota, nutraceuticals, natural compounds, methotrexate toxicity, toxicity management

## Abstract

There are multiple concerns associated with methotrexate (MTX), widely recognized for anti-neoplastic and anti-inflammatory effects in life-threatening disease conditions, i.e., acute lymphoblastic leukemia, non-Hodgkin’s lymphoma, psoriasis, and rheumatoid arthritis, due to long-term side effects and associated toxicity, which limits its valuable potential. MTX acts as an inhibitor of dihydrofolate reductase, leading to suppression of purine and pyrimidine synthesis in high metabolic and turnover cells, targeting cancer and dysregulated immune cells. Due to low discrimination between neoplastic cells and naturally high turnover cells, MTX is prone to inhibiting the division of all fast-dividing cells, causing toxicity in multiple organs. Nutraceutical compounds are plant-based or food-derived compounds, used for their preventive and therapeutic role, ascertained in multiple organ dysfunctions, including cardiovascular disease, ischemic stroke, cancer, and neurodegenerative diseases. Gut microbiota and microbiota-derived metabolites take part in multiple physiological processes, their dysregulation being involved in disease pathogenesis. Modulation of gut microbiota by using nutraceutical compounds represents a promising therapeutic direction to restore intestinal dysfunction associated with MTX treatment. In this review, we address the main organ dysfunctions induced by MTX treatment, and modulations of them by using nutraceutical compounds. Moreover, we revealed the protective mechanisms of nutraceuticals in MTX-induced intestinal dysfunctions by modulation of gut microbiota.

## 1. Introduction

Antifolate drugs represent an old class of pharmaceuticals which interfere with folate metabolism and have been used to treat proliferative disorders, e.g., acute lymphocytic leukemia, breast cancer, and parasitic, and microbial diseases [1,2,3].

Methotrexate (MTX), an antimetabolite of folic acid, which acts as an inhibitor of dihydrofolate reductase (DHFR), exhibited anti-neoplastic and immunomodulatory effects in the area of malignant and non-malignant diseases [4]. Though the mechanisms behind its anti-inflammatory effects against psoriasis, rheumatoid arthritis (RA) and anti-cancer effects upon blood malignancies and other neoplasia are quite understood, the precise therapeutic effect of MTX is not clarified [5]. 

Although MTX therapy attracted attention in preclinical and clinical settings of numerous inflammatory and cancer disorders, its associated adverse effects and the toxicity on multiple organs related to MTX remain an important concern and a cause of drug withdrawal [6,7].

Nutraceutical-based therapy, first described by DeFelice in 1989, refers to food-based compounds which exhibited multifaced roles in preventing and treating life-threatening diseases [8,9]. Nutraceutical compounds are represented by a wide range of nutrients and phytochemicals, such as dietary bioactive peptides and lipids, fat soluble vitamins, amino-acids, micronutrients [10]. Some of the most common nutraceuticals, such as resveratrol [11], curcumin [12], coenzyme Q10 (ubiquinone), α-lipoic acid [13], β-carotene [14], quercetin [15] have been shown to alleviate oxidative stress, and apoptosis-induced cellular injury, targeting pro-inflammatory signaling pathways, by acting on nuclear and mitochondrial targets [16,17].

Growing evidence suggested the protective effects of nutraceuticals in multiple in vitro and vivo disease models, including ischemia–reperfusion injury [18], cardiovascular diseases [19], ischemic stroke [20], cancer [21], neurodegenerative diseases [22], and inflammatory bowel diseases [23].

Within MTX therapy, preclinical and clinical studies reported long-term side effects and toxicity effects on multiple organs and a promising therapeutic strategy aimed at restoring the toxicity of MTX is currently needed. The negative effects of MTX treatment are reflected on multiple organs, including hepatic fibrosis, acute lung injury, dysregulation of gut microbiota and nephrotoxicity [24,25,26,27]. There are a wide range of short- and long-term side effects, ranging from nausea, drowsiness, liver enzymes elevation, to renal insufficiency, hepatic fibrosis/cirrhosis, pulmonary fibrosis and life-threatening blood disorders, i.e., pancytopenia and aplastic anemia [5].

MTX toxicity and MTX side effects restrict its clinical uses to moderate and severe forms of disease or drug-resistant diseases [28]. Thus, finding promising natural components aimed at ameliorating toxicity and side effects of MTX represents a future direction in MTX therapy. In MTX acute nephrotoxicity, the management is focused on fast intravenous fluid hydration, urine alkalinization, and rescue of folic acid [29].

Multiple preclinical studies explored the anti-inflammatory and immunomodulatory effects of several Nutraceuticals in liver and kidney injury, acute lung injury and gut microbiota dysregulation induced by treatment with MTX [30,31,32]. An in-depth overview of the mechanism of action of nutraceuticals in experimental MTX toxicity models could provide further directions in MTX therapy. This review aims to overview the main nutraceuticals involved in the beneficial effects against MTX-induced organ injuries and to propose promising nutraceuticals as adjuvant therapy in patients treated with MTX.

## 2. MTX as a Disease-Modifying Agent

DHFR is a crucial enzyme involved in the reductive process of dihydrofolic acid to tetrahydrofolic acid, an essential oxido-reductive process required for the de novo synthesis of purines, pyrimidines, and certain amino acids [33]. These chemical components are essential for cell proliferation and cell growth [33].

MTX is an antimetabolite of folic acid, which was first approved by FDA for treating only life-threatening malignancies, treatment-resistant or treatment non-respondent forms of psoriasis and RA, and also those forms with severe course of disease [4]. The mechanism of action underlying anti-neoplastic and immunomodulatory effects of MTX is based on inhibition of DHFR, thus promoting suppression of purine and pyrimidine synthesis in cell proliferation [34,35].

MTX binds to the same active site of DHFR, with a 1000-fold increase in the affinity of DHFR over that of dihydrofolic acid [36]. Binding of the MTX to its substrate, DHFR, acts in a NADPH-dependent manner [36,37]. This dependency could also play a critical role for the selective toxicity of MTX within malignant cells, malignant cells revealing a higher NADPH/NADP ratio compared to the intact and viable cells [36,38].

However, the anti-inflammatory mechanism of MTX does not proceed by inhibiting DHFR enzyme and in-depth mechanistic data of anti-inflammatory features are still needed. Mounting experimental studies proposed several mechanisms of action of MTX treatment, including counteracting reactive oxygen species (ROS) production [39,40,41], inhibition of pyrimidine pathway enzymes [42], release of adenosine [43,44], and regulation of cytokine production [45,46].

Multiple ways of administration, i.e., orally, intravenously, intramuscularly, or intrathecally allow a good bioavailability and an effective mechanism of action of MTX in multiple pathological contexts [47].

After oral administration, it is actively absorbed within proximal jejunum, the enteric transport being mediated by proton-coupled folate transporter (PCFT/SLC46A1) [5]. Even in the absorption phase, a small percentage of 5% of MTX is metabolized to 4-amino-4deoxy-N10-methylpterrroic acid, an inactive metabolite of MTX [5].

Within the cell membrane, the cellular uptake and efflux are mediated by specific transporters, including reduced folate carrier (RFC1), the proton-coupled folate transporter (PCFT), and ATP-binding cassette proteins (ABCC) [5]. The bioavailability ranges from 30% to 90% [5,48], varying widely among different subtypes of patients and it decreases with increasing dose, suggesting the saturation of the active transporters with MTX [49,50].

The distribution of MTX to the body’s tissues mainly depends on the reduced folate carrier 1 (RFC1), involved in the transport of reduced folates such as 5-methyl THF [51]. Once distributed to cells and tissues, MTX is rapidly converted to MTX polyglutamates (MTX-PG) by folylpolyglutamate synthase, which binds six glutamate residues to MTX, therefore sustaining its intracellular retention and increasing enzyme binding affinity [52]. Some MTX is hydrolyzed to 7-hydroxymethotrexate by aldehyde oxidase in the liver [53].

Renal excretion constitutes the primary elimination route, mainly in the intact form (more than 80%) and 3% as the 7-hidroylated form [54]. MTX is recycled by enterohepatic circulation, about 8% and 26% of plasmatic MTX being excreted in the bile [52,55,56]. The clinical use of MTX ranges from neoplastic diseases, such as acute lymphoblastic leukemia, acute promyelocytic leukemia, non-Hodgkin’s lymphoma, to epidermoid cancers of the head and neck, early-stage breast cancer, osteosarcoma and several types of gestational trophoblastic neoplasia [57,58].

In addition to anti-cancer effects, MTX poses anti-inflammatory and immunomodulatory effects. Inflammatory diseases, such as inflammatory bowel diseases, vasculitis, systemic lupus erythematosus, multiple sclerosis, transplantation surgeries could benefit from MTX therapy [59,60]. As a disease-modifying agent, MTX is used for treating RA, juvenile idiopathic arthritis (JIA), and psoriasis [57,58,61].

Despite its widespread clinical use and usefulness, MTX comes with pitfalls, including short- and long-term side effects such as hepatotoxicity, nephrotoxicity, and leukopenia, which can predispose patients to severe infections [57,61]. An overview of the main pharmacological features of MTX as a therapeutic agent in non-malignant and malignant diseases is provided in Table 1.

## 3. Gut Microbiota-Related Changes following Treatment with MTX

### 3.1. Involvement of Gut Microbiota in Health and Disease

Microbiota is part of our human microecosystem, which poses multiple regulatory roles in our body in both health and pathological conditions [63]. Widely distributed within the organism, microbial species colonize multiple organs and cavities, i.e., oral cavity, gut, lung, skin, vagina, etc. [64]. Microbiota consists of a large abundance and density of microorganisms, including bacteria, fungi, viruses, protozoa, and archaeal, which live in symbiotic or parasitic relationships [64,65].

With the greatest diversity and abundance, the gut microbiota harbors an extensive community of over 100 trillion microbial cells, with a 150-fold increase in the gut-regulatory genes compared to human genome [66,67]. The two most dominant intestinal phyla include *Firmicutes* and *Bacteroidetes*, with *Lactobacillus*, *Faecalibacterium*, *Clostridium*, *Enterococcus* accounting for dominant genera of *Firmicutes* and *Bacteroides*, and *Prevotella*, reaching the most of *Bacteroidetes* genera [68].

Gut microbiota and its derived metabolites exert multiple essential roles in the body, from immune modulation, to metabolic, digestive functions and biosynthesis of active compounds [63,69]. Compositional changes in gut bacterial species have been reported in large spectrum of disease conditions, i.e., cardiovascular dysfunctions [70], stroke [71], neurodegenerative and cognitive disorders [72,73], cancer-related disease and autoimmune disorders [74,75]. Aging, diet, smoking and patient-associated comorbidities, i.e., diabetes, obesity are influencing factors with a decisive impact on gut bacterial profile [76,77,78,79].

### 3.2. The Role and Protective Effects Exhibited by Microbiota

Gut microbiota interacts in a bidirectional manner with multiple organ systems, influencing each other; systemic changes contribute to intestinal dysbiosis, and also intestinal microbiota dysregulation is involved in disease pathogenesis and organ dysfunction [80]. Evolving research studies evaluated the relationship between dysregulation of gut microbiota and dysfunctions of other organ systems, in relation with disease pathogenesis and therapeutic insights. Referring to this interaction, they are classified as the gut–liver axis [81], the gut–brain axis [82], and the gut–liver–brain axis [83].

Gut microbiota exerts immunomodulatory and anti-inflammatory functions and interact in a bidirectional manner with multiple organ systems through its key mediators, microbiota-derived metabolites, specifically short-chain fatty acids, (SCFAs) [84]. SCFAs, consisting of *acetate*, *propionate*, and *butyrate* are carboxylic acids, formed by chains of 2–6 carbon atoms, which are produced by anaerobic bacterial fermentation of complex dietary carbohydrates within intestinal lumen [85]. The source of biosynthesis is represented by dietary fibers such as plant cell wall-derived polysaccharide, soluble oligosaccharide, and also endogenous molecules, such as mucin [85,86]. Different microbial taxa are responsible for SCFAs synthesis: acetate formation is mostly mediated by enteral bacteria, such as Prevotella spp., Bifidobacterium spp., Bacteroides spp., Clostridium spp., Ruminococcus spp., and Streptococcus spp. [87]; propionate formation is regulated by few bacterial genera, *Salmonella enterica* serovar Typhimurium and *R. inulinivorans* [88,89]; and dominant species responsible for butyrate formation are *Coprococcus*, *F. prausnitzii*, *E. hallii*, *E. rectale*, *Ruminococcus bromii* [90,91].

After synthesis in the intestinal lumen, the cellular uptake of SCFAs proceeds via specific transporters expressed on epithelial cells of the small intestine and colon, in a manner dependent on Na^+^/H or Cl/HCO_3_ co-transporters [86]. The highest SCFAs concentration is reached in the cecum, followed by descending colon and ileum, in relation to biodiversity and composition of microbial species [92]. After entering into intestinal epithelial cells, SCFAs exert regulatory roles by interacting with specific receptors, G protein-coupled receptors (GPCR) and or histone deacetylases (HDACs) [93,94,95]. At the nuclear level, propionate and butyrate induce transcriptional regulation and post-translational modification of histones, by targeting lysine and histone deacetylase (K/HDAC) [86]. 

SCFAs could interact with different immune cells, contributing to innate and adaptive immune homeostasis [84,93,94,95]. SCFAs, acetate or propionate stimulate bone marrow hematopoiesis, along with activation of Th cells differentiation and increasing expression of specific chemoattractant molecules on immune cells [96,97]. Local or systemic immune responses are mediated by SCFAs, butyrate inhibiting pro-inflammatory pathways, such as NF-κB, thus decreasing pro-inflammatory cytokines, i.e., TNF-α, IL-6, IL-12 and activating anti-inflammatory cytokines, i.e., IL-10 [92].

Changes in the abundance and diversity of gut microflora species, known as “dysbiosis”, are reported in multiple intestinal inflammatory dysfunctions, providing high value as theragnostic tools in disease settings [98]. Evolving research studies depicted metabolomic and microbiome profiling data of serum and fecal patient samples, giving insights into bacterial-based biomarkers as promising predictors of therapeutic response and clinical outcome [99,100].

### 3.3. The Role of Microbiota for Promoting an Intact Epithelial Cell Barrier

Intestinal stem cell niches (ISCN) are at the basis of epithelial barrier renewal and maintaining of intestinal barrier integrity [101]. By sustaining proliferative and differentiation processes in a dynamic manner, ISCNs give rise to specialized epithelial cells: enterocytes, enteroendocrine cells, Paneth cells, microfold (M) cells and goblet cells [102]. Epithelial cell surface expresses specific receptors, namely pattern recognition receptors (PRRs), which bind to microbial ligands expressed on enteric commensal bacteria to maintain intestinal epithelial homeostasis against pathogen bacteria species and other intestinal insults [103,103]. Therefore, bacterial molecules and its associated metabolites are recognized by several PRRs, consisting of Toll-like receptors (TLRs), which are expressed on epithelial and immune cells, aimed at protecting and immunomodulating intestinal surface barrier [103,103]. Specific conserved motifs in epithelial cell receptors, including TLR/MyD88 and Nucleotide oligomerization domain (NOD)-like receptors (NLRs), exhibited protective roles against invasion of gut microbiota species of epithelial cells, by synthesis of antimicrobial factors, such as defensins and cathelicidins [103,104,105]. Inflammatory responses triggered by commensal bacteria are prevented by sequestration of microflora by mucosal epithelial cells, preventing the “deleterious” activation of TLRs by beneficial microflora [106,107]. In a model of chemical colitis of pathogen-free mice, Rakoff-Nahoum et al. found that activation of TLR2 and TLR4 by enteric commensal bacteria is necessary for protection upon mucosal injuries and mice-related mortality [103]. In addition to protective effects of epithelial cell barrier, PRRs by interacting with microbial components promoted pathways involved in cellular proliferation [105].

The breakdown of intestinal barrier integrity is involved in multiple intestinal dysfunctions, such as irritable bowel syndrome, metabolic syndrome, inflammatory bowel diseases, and necrotizing enterocolitis, and obesity [108,109,110]. The functional state of intestinal barrier function is a hallmark of gut homeostasis, depending on multiple influencing factors, (e.g., cellular, biochemical, immunological, and bacterial) [105]. Commensal gut bacterial species sustain both maintenance and/or restoring processes of epithelial gut barrier, by promoting cellular processes of differentiation, proliferation and migration and survival [111,112]. SCFAs elicited several modulatory roles to maintain an intact intestinal epithelial barrier at different levels.

Butyrate constitutes the main energy source of colonocytes, contributing to their structural integrity [86]. SCFAs by interacting with GPCRs, (e.g., butyrate-GPR109a, acetate, propionate, butyrate—GPR43, GPR41) induce gene expression regulation and signaling transduction, promoting epithelial cell differentiation, apoptosis, and proliferation [113]. SCFAs sustain epithelial barrier integrity, by reducing epithelial permeability (e.g., modulation of HIF, STAT3 signaling pathways) [86], regulating tight junction (TJ) proteins [105], and mucus layer thickness (e.g., targeting MUC expression) [114] and promoting antimicrobial peptides synthesis [115].

The distinctive features of the intestinal surface, such as the “intact” barrier and, at the same time, being selective and dynamically permeable, are conferred by intercellular connections, consisting of TJs, adherens junctions, the desmosomes, and the gap junctions [116,117]. Gut microbiota bacterial species modulate expression of several intercellular connections, therefore maintaining intestinal epithelial barrier homeostasis. In Caco-2 and HT-29 cells cultured on human milk oligosaccharides, *B. infantis* and *B. bifidum* regulated TJs proteins such as ZO-1 and occludin expression, whereas *L. rhamnosus* and *Faecalibacterium prausnitzii* modulated Occludin and E-cadherin expression to alleviate impaired gut barrier function in a mouse model [118,119].

### 3.4. The Influence of MTX on Gut Microbiota

The first experimental report describing the response of small-intestine epithelial cell under MTX was mentioned by Taminiau et al., in 1980 [120]. The authors reported that, under an intravenous dose of 30 mg/kg for 24 and 48 h, MTX exhibited suppressive effects on cellular mitoses in crypts, decreased intestinal villi, and also inactivated thymidine kinase activity, an essential enzyme within epithelial cells from intestinal crypts [120]. In a metabolomic and microbiome profiling study conducted in male Sprague Dawley rats treated with MTX, authors depicted changes in fecal samples metabolites and compositional bacterial changes at different time-points after MTX treatment [26]. Changes in the metabolomic profile of fecal samples, specifically 2,4-diamino-N(10)-methylpteroic acid (DAMPA) concentration in faces have been positively correlated with abundance of *Prevotellaceae*, *Anaeroplasmataceae*, *Lactobacillaceae* and *Ruminococcaceae*, and negatively correlated with bacterial species of *Deferribacteraceae* and *Coriobacteriaceae* [26]. Moreover, urine sample of rats exhibited methionine sulfoximine metabolites, which was associated with *Ruminococcaceae* species at 48 h. Bacterial abundance of *Prevotellaceae* and *Anaeroplasmataceae* was associated with two metabolites under MTX treatment, fecal glutamate and urine 5-hydroxyindole acetic acid at 48 h. Up to 24 metabolic compounds, i.e., dipeptides, tripeptide, organic acids, have been shown to be altered in fecal samples of rats upon MTX treatment in a dose-dependent manner [26]. Gut microbiota of rats exhibited changes in bacterial species after MTX treatment, with an enrichment of the abundance of Firmicutes over the Bacteroidetes, when treating rats with low doses, with an reverse trend at high doses [26]. Changes of gut microbial signature of rats under MTX treatment were driven by an increase in *Peptostreptococcaceae* and *Porphyromonadaceae* and a decrease in the relative abundance of *Ruminococcaceae*, *Erysipelotrichaceae* [26]. Alteration in gut microbial composition has been revealed in MTX-induced liver injury, which was restored after 40 mg/kg of Magnesium Isoglycyrrhizinate (MgIG) supplementation [32]. Under a long course of 30 days of MTX treatment, gut microbiota in mice exhibited an increase in bacterial proliferation of *Muribaculaceae*, and also a decrease in *Lactobacillus* abundance [32]. Within colon permeability, MTX treatment induces alteration in expression of TJs, i.e., ZO-1 and Claudin-1 and cell adhesion protein, E-cadherin, suggesting the impact of dysregulated gut microflora on the expression of intercellular connections [32]. MgIG could reverse the dysregulated expression levels of these proteins induced by MTX treatment, mitigating epithelial changes in leaky gut [32]. 

In addition to the alteration of gut microbiota induced by MTX, bacterial changes in gut microflora occurred during RA, for which MTX is indicated, suggesting the influence of disease progression on gut microbiota and further influencing MTX efficacy. In 212 fecal samples of RA patients, the bacterial proliferation of *Haemophilus* spp. was decreased, being negatively correlated with serum autoantibodies levels, whereas RA patients exhibited an enrichment of *Lactobacillus salivarius* [121]. By DNA sequencing of stool samples of 29 children with JIA, Öman et al. depicted distinct signature of gut microbiota composition upon MTX treatment, with an increase in *Subdoligranulum* and under-representation of *Rikenellaceae*, *Veillonellaceae*, *Bacteroidales_S24-7_group* [122]. Moreover, the SCFAs levels differ in MTX group compared with Etanercept group, with an increase level of iso-butyrate in fecal samples of 15 JIA patients treated with MTX [122]. Multiple experimental studies examined the potential of pre/probiotics, vitamins, plant-based extracts to alleviate structural changes in the intestine architecture upon difference disease models [123]. Changes in the bacterial profile of gut microbiota upon MTX treatment suggest the role of adjuvant therapies in restoring the intestinal bacterial balance, nutraceutical compounds being promising candidates. 

Several bacterial changes in gut microbiota of animal models or patients treated with MTX are viewed in Table 2.

## 4. Nutraceuticals Use to Counteract MTX Toxicity in Experimental Models

**Figure 1:**
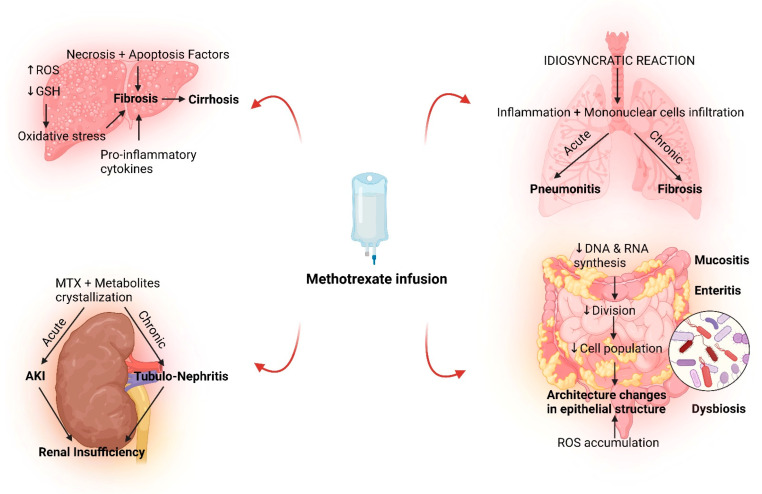
The main mechanisms of MTX toxicity in experimental disease models of organ injury. Several organ dysfunctions have been reported following MTX treatment: MTX-induced liver toxicity represented by fibrosis and cirrhosis, MTX-associated lung dysfunction consisted of pneumonitis and fibrosis, kidney injury related to MTX treatment, including acute kidney injury and tubulo-nephritis, intestinal changes upon MTX including mucositis, enteritis, and dysbiosis. Abbreviations: AKI, acute kidney injury; GSH, glutathione; ROS, reactive oxygen species.

Given the plethora of side effects, both acute and chronic, the management of MTX toxicity needs to incorporate strategies aimed to prevent the side effects associated with long-term use and adjusting treatment plans for acute toxicity [5,47]. The schematic bellow (Figure 1) represents an overview of the main molecular and cellular mechanisms involved in MTX multi-organ toxicity according to experimental models.

Preventing the toxicity associated with long-term use could be achieved by using the lowest effective dose, monitoring the blood concentration of MTX by using commercially available immunoassays [125], and co-treatment of MTX with natural compounds which can prevent buildup of MTX, slow down or prevent side effects.

Although the therapeutic effect is obtained by administering the effective dose therapy [47,125], dose individualization considering both the severity of disease and the MTX metabolic rate could prevent toxicity events [47,61].

Therapeutic drug monitoring (TDM) has become a staple for many therapies that utilize drugs with a narrow therapeutic index such as MTX. TDM can be used for two reasons in MTX treatment: to ensure that MTX concentration is high enough to be therapeutically effective, and to minimize dose-dependent toxic events [47,126,127]. MTX monitoring can be performed in a clinical setting using commercially available immunoassay kits, thus minimizing the difficulty and time requirement of such operation [125].

Several combined therapeutic strategies aimed at preventing both acute toxicity and side effects associated with long-term use have been recently proposed. One such example is the use of folic acid or folinic acid (5-formyl derivative of tetrahydrofolic acid, Leucovorin) supplements in order to prevents folate depletion for healthy tissue, and thus prevent hematologic side effects [127,128], but also allow the use of higher doses of MTX in order to achieve a better therapeutic result without significantly increasing the chance for adverse events [125,127]. Another example is the use of urine alkalinization compounds, in order to increase the excretion of MTX and prevent nephrotoxicity [127,128]. Moreover, urine alkalinization can be used for episodes of acute renal toxicity that result in acute kidney insufficiency (AKI) together with glucarpidase (a recombinant bacterial carboxypeptidase G2). Creating a pH of 7.5 will reduce the amount of MTX and its 7-hydroxilated form crystalizing, further improving renal excretion, while glucarpidase will cleave MTX into DAMPA and glutamate, two non-toxic metabolites, thus rapidly lowering MTX plasma concentration in patients with AKI [127,128,129].

Nutraceuticals are defined as plant or food-based compounds generally sold in medicinal forms, for both preventive and therapeutic roles [8,9]. Additionally, the beneficial roles in physiological or disease conditions for providing protection against chronic disease have been demonstrated in multiple preclinical and clinical studies [130]. Under this definition several items are covered, such as vitamins, minerals, herbs and other botanicals extracts, to amino acids, prebiotics, probiotics, and other dietary substances [131]. A considerable amount of the current research into nutraceuticals focuses on plant-based therapeutics, through the use of concentrates and purified extracts, due to the large variety of active compounds found in plants [131,132].

Emerging preclinical and clinical studies ascertained the role of nutraceuticals in multiple disease contexts, including cardiovascular diseases, diabetes, cancer, allergies, and visual disorders [133,134,135,136,137,138,139,140]. Through these studies nutraceuticals make themselves out as a viable and simple method not only of treatment, but also of prevention of certain diseases.

Multiple chemotherapeutical agents frequently used in cancer therapy proved to have multi organ long-term side effects and toxicity that hinders their therapeutic efficacy and the life expectancy of oncologic patients [125,141,142,143,144,145,146,147,148]. These pitfalls of chemotherapeutic agents prompt the need for therapeutic strategies aimed at reducing the toxicity of cancer drugs. Plant-based therapies have been established as a protective measure in cancer therapy toxicity models [149,150,151].

MTX is part of those chemotherapeutical agents to have multi organ long-term side effects, with studies showing significant hepatotoxicity, nephrotoxicity, and potential pulmonary fibrosis as some of the most dangerous adverse effect after employing MTX [127,128], and list them as a common cause of therapy withdrawal [47,57,61,128]. Thus, MTX could benefit from nutraceutical co-treatment in order to reduce or prevent certain adverse events.

### 4.1. Hepatotoxicity

The underling molecular mechanism behind MTX-induced hepatotoxicity is not yet completely understood; however, considerable experimental and clinical evidence proposed oxidative stress as a contributing factor through the increased generation of ROS, alongside with decreased antioxidant defense systems [152,153,154].

Prevention of MTX-induced hepatotoxicity using nutraceuticals has been extensively studied, consisting in a wide variety of nutraceutical categories, including vitamins, nutrients and dietary supplements, with varying levels of effectiveness [155,156,157].

In an experimental mouse model of MTX toxicity, pre-treatment with epicatechin at doses of 25, 50 and 100 mg/kg have been shown to have protective effects. The beneficial effects elicited by epicatechin compound might be explained by improving the antioxidant defense system, posing anti-inflammatory effects, and alleviating histopathological changes [30]. In another experimental model, treatment with thymoquinone at a dose of 10 mg/kg/day for 10 days has been shown to have protective effects in rats treated with MTX, by regulating antioxidant, anti-nitrosative, anti-inflammatory, and antiapoptotic mechanisms [158].

An amount of 50 and 100 mg/kg/day of ferulic acid has been shown to have protective effects in MTX-induced mice model of oxidative stress injury [159]. 

Rhein treatment in rats proved to be another protective measure against MTX-induced hepatotoxicity, as reported by Bu et al. [160]. The underling mechanism of this effect was linked to the upregulation of several signaling pathways, such as nuclear factor (erythroid-derived 2) like 2 factor (nrf2), B-cell lymphoma-2 (Bcl-2), heme oxygenase 1 (HO-1), and glutamate-cysteine ligase catalytic subunit (GCLC) [160].

Gossypin, an anti-tumoral and anti-inflammatory nutraceutical, was examined by Mohamed et al. in a rat model of MTX toxicity. The anti-inflammatory mechanism responsible for the reduction in inflammatory cell infiltration of the liver proceeds by inhibiting the TGF-β/NFκ-B signaling pathway [161].

Berberine is a plant-based nutraceutical that has been extensively tested in multiple disease models for its therapeutic effects. Using a MTX-induced hepatotoxicity model in rat model Mehrzadi et al. [162] analyzed the protective effects of berberine pre-treatment, using 100 mg/kg doses for 10 days. Berberine extract proved to have hepatoprotective effects by activating antioxidant defense enzymes such as GSH and GPx [162].

Resveratrol’s effectiveness as a protective agent in MTX hepatotoxicity was analyzed and confirmed in two different animal studies. Tunali-Akbay et al. [163] reported a neutrophil-dependent antioxidant mechanism through which resveratrol enacts its hepatoprotective effects, while Dalaklioglu et al. [152] reported the inhibition of lipid peroxidation through scavenging of superoxide and hydroxyl radicals by resveratrol.

Similar to resveratrol, gallic acid inhibits lipid peroxidation through the scavenging of ROS [164]. Jafaripour et al. [165] reported that inhibition of lipid peroxidation was behind the action of another nutraceutical, rosmarinic acid, which was able to mitigate the oxidative stress induced by MTX.

The effectiveness of two plant extracts widely used in traditional medicine, turmeric and Ginkgo biloba extract, was evaluated in two separate studies using rat model. Experimental data of Ginkgo biloba extract revealed a dose-dependent protective effect of the nutraceutical [166]. The beneficial effect might be explained through the modulation of the innate antioxidative mechanisms such as glutathione and glutathione S-transferase [166]. Moghadam et al. [167] reported similar findings while evaluating the effectives of turmeric extracts, with the added benefit of scavenging of ROS, and thus reducing lipid peroxidation.

Ahmad et al. [168] described the hepatoprotective effect manifested by sinapic acid through the regulation of the nrf2/heme oxygenase 1 (HO-1) and NF-κB signaling pathways. By modulating both oxidative enzymes, such as malondialdehyde, nitric oxide and catalase, and antioxidative systems, i.e., glutathione peroxidase, glutathione reductase activity, naringin was able to provide significant protection against MTX-induced oxidative stress and preserve the histological structure of rat hepatic tissue [169].

Rutin is a glycoside flavanol found in several plants, including citrus plants [170], which was previously demonstrated to exert a hepatoprotective effect against other chemotherapeutical agents [171,172] Erdogan et al. [173] reported that rutin, by elevating tissue superoxide dismutase and plasma glutathione peroxidase, showed similar hepatoprotective effects against MTX toxicity.

Propolis and propolis extracts have been examined as nutraceutics as well. Çetin et al. [174] reported the beneficial effects of propolis in preventing MTX hepatic injury in rats. Chrysin, a flavonoid extracted from honey and propolis, has been reported to alleviate oxidative stress and apoptosis induced by MTX in rats [175].

Two dietary supplements with promising effects have been tested for their supposed protective effects, indole-3-carbinol and alfa lipolic acid. Indole-3-carbinol, by upregulating antioxidant defense systems, alleviates MTX-induced hepatic injury [176]. On the other hand, alfa lipolic acid was able to prevent MTX-induced hepatotoxicity through the scavenging of ROS [157]

Vitamin supplements have been tested as well, with various results. Akbulut et al. [156] demonstrated the limited protective effect of ascorbic acid compared to other hepatoprotective agents in MTX-induced toxicity. In rat model of MTX-induced liver injury therapeutic delivery of β-carotene, an important source of vitamin A in the human diet, with antioxidant properties, led to decreased hepatic MDA activity and increased hepatic SOD, CAT and GSH peroxidase activities under conditions of [153]. Amirfakhrian et al. [177] showed the protective effect of vitamin E on liver architecture assessed by 99mTc-phytate functional imaging. Ismail et al. [178] report that while thiamine pyrophosphate exhibited protective effects against MTX hepatotoxicity, thiamine alone was ineffective. The beneficial effect was attributed to a reduction in MTX-induced NADP inhibition. 

Dietary deficiencies were also reported to influence the severity of MTX hepatotoxicity [155]. A choline deficient diet resulted in an increase in the extent of fatty infiltration in rats treated with MTX as compared to normal rats treated with MTX, outlining the importance of choline in preventing MTX-induced fatty liver injury.

### 4.2. Nephrotoxicity

The most commonly described mechanism of MTX nephrotoxicity is the precipitation of MTX and its metabolites in the renal tubules [127]. Other mechanisms have also been proposed to play a concomitant role, such as constriction of the afferent capillary and direct effects on the mesangial or tubular epithelial cells [179,180].

Because the primary elimination route of MTX is renal excretion, with a high likelihood of nephrotoxicity, preventing those adverse events has been attempted through the use of various nutraceutical compounds.

Aladaileh et al. [181] have described the mechanism behind formononetin nephroprotective properties. Through the upregulation of nrf2/HO-1, formononetin successfully prevented MTX-induced renal injuries in a rat model. A similar molecular mechanism was found by Hassanein et al. [182] when studying the effectiveness of berberine as a nephroprotective agent. Rosmarinic acid, a polyphenolic nutraceutical compound, was reported to regulate the same pathway as berberine and formononetin, having a similar effect in rat models [165].

Paeonol and paeoniflorin are two aromatic compounds found in plants from the genus *Paeonia* spp. [183,184,185]. Morsy et al. [186] reported how paeonol administration increased the expression of the renal efflux transporter P-glycoprotein, which accelerates MTX elimination, limiting the nephrotoxic effect of the drug. The protective effect of paeoniflorin-6′-O-benzene sulfonate against MTX toxicity proceeds by targeting expression apoptotic proteins, such as Bax, cleaved-caspase-3, and cleaved-caspase-8 [187].

In an experimental model of MTX nephrotoxicity in rats, Elsawy et al. [188] observed the protective effects of naringin at doses of 20–40 and 80 mg/kg. 

In another experimental model, Oguz et al. [189] evaluated the effectiveness of lycopene alone and in combination with melatonin. Both therapeutic regimes provided significant reduction in TNF-α, interleukin 1-beta (IL-1β) and ceruloplasmin levels. Further histopathological evaluation of renal tissue revealed a superior effect for the combined regime, with both schemes showing a significant protective effect against MTX-induced histological changes.

Inhibition of lipid peroxidation and increase in antioxidative status of cells occurred as a result of treatment with caffeic acid phenethyl ester at a dose of 10 mmol/kg in MTX-induced oxidative stress in rat kidney [190].

The effectiveness of gallic acid as a nephroprotector was assessed in two different animal studies. Asci et al. [191] and Olayinka et al. [164] have reported that gallic acid, through the reduction in oxidative stress, was effective at preventing renal injury induced by MTX. 

Curcumin, primarily found in turmeric powder, is reported to have antioxidant properties, thus posing nephroprotective effects in rat models for MTX toxicity [192].

Quercetin, in a 15 mg/kg/day for 5 days, has been shown to lessen the degenerative changes and reduce apoptosis in rat kidney upon MTX treatment. Additionally, quercetin increased superoxide dismutase, glutathione peroxidase, and catalase levels, effectively reducing oxidative stress induced by MTX [193].

Several plant extracts have been tested for nephroprotective effects against MTX toxicity. Sherif et al. [194] have tested Ginkgo Biloba extracts, finding that it decreased renal TGF-β mRNA and MALAT1 expression, and regulated PI3K/Akt/mTOR signaling. Nigella sativa oil increased glutathione levels and prevented histological changes of renal tissue in a prolonged exposure of animal model [195]. Pre-treatment and co-treatment of MTX with garlic extracts in a rat model proved to be effective at preventing renal injury by increasing renal antioxidant enzyme activity [196]. Abouelela et al. [197] report the use of Ceiba pentandra extract to prevent MTX-induced renal toxicity in rats. The effects might be explained by improved renal antioxidant capacity and reduced MTX-induced oxidative stress. Hydro-Alcoholic extract of raspberry fruits has been shown to protect against MTX nephrotoxicity by upregulating the expression of aquaporin 1 in a dose-dependent matter [198]. Polyherbal combinations have been teste as well, with Sharma et al. [199] reporting on the use of Roots of B. diffusa and R. emodi, flowers of N. nucifera and stem bark of C. nurvala concomitantly. Histopathological analysis showed an alleviation of rat renal injury caused by MTX, when rats were treated with the polyherbal mixture, an effect explained by ROS scavenging and by improved renal antioxidant capacity.

### 4.3. Gastrointestinal Toxicity

Studies have shown that ROS production plays a key role in the mechanism behind gastrointestinal mucositis and enteritis caused by MTX treatment [200,201]. By having a low discrimination ability between tumor cells and fast-dividing cells and inhibiting DNA and RNA synthesis of fast-dividing cells, MTX counteracts epithelial intestine cells with a high turnover [120,202]. This results in the inhibition of division, a decrease in cell population, and ultimately leads to architectural and functional changes of intestinal epithelium.

Turmeric extracts, and mainly curcumin, have been reported to have various protective effects against MTX-induced toxicity. Song et al. [203] reported protective effects of curcumin against MTX aggression against intestinal mucosa. The beneficial effect might be explained by activation of mitogen-activated protein kinase phosphatase-1 and antioxidative mechanisms of superoxide dismutase and repression of NF-κB [203].

The effect of glutamine supplementation in MTX-induced enteritis has been studied on multiple animal models. in rat models, glutamine reduced intestinal injury, improved nutritional status, decreased bacterial translocation by preserving intestinal mucosa integrity, and improved survival rate [204]. However, in a cat model, glutamine supplementation was unable to preserve intestinal function [205].

Gastroprotective effects of several plant extracts have been tested against multiple animal models of gastrointestinal toxicity caused by MTX treatment. Shi et al. [206] explored gastroprotective effect of steamed root of Rehmannia glutinosa Libosch in intestinal mucositis model of MTX in a rat model. The extract mitigates MTX intestinal injury by alleviating oxidative stress and inflammatory responses [206]. Paullinia cupana, by increasing antioxidant systems and inhibiting IL-1β, has been shown to preserve intestinal integrity against MTX toxicity [207]. Wang et al. [208] have tested the effectiveness of glycyrrhizin acid, constituent of Glycyrrhiza glabra root, against MTX-induced enteritis. By suppressing the NF-κB and MAPK signaling pathways, glycyrrhizin acid showed significant protective effects against enteritis caused by MTX treatment in rats. Albiflorin is a glycoside isolated from the same plant as paeoniflorin, which has anti-inflammatory properties. Zhang et al. [209] reported immunomodulatory and anti-inflammatory features of albiflorin by inhibiting NF-κB/NLRP3 pathway, and also significantly reducing oxidative stress in MTX-induced enteritis. 

Fatty acids and fatty acid derivates have been experimentally investigated as potential protective agents against oral mucositis, gastric mucositis and loss of intestinal integrity. Alfa lipolic acid, a naturally occurring caprylic acid derivative, has been tested as a preventive agent against oral mucositis and oxidative stress induced by MTX in rats [210]. Through the increase in glutathione and superoxide dismutase, and inhibition of apoptosis, alfa lipolic acid attenuated MTX-evoked alterations of the intestinal wall [210]. da Silva Ferreira et al. [211] reinforced the potential prophylactic benefits of butyrate, through the use of a novel model using 3D intestinal organoids derived from mouse ileum. The anti-inflammatory and anti-apoptotic properties of omega 3 fatty acids was reported to limit the intestinal damage in rats treated with MTX [212]. The molecular mechanism relies on downregulation of NF-κB, ciclooxigenase-2 and TNF-α. 

Yilmaz et al. [213] reported the beneficial effect of daily use of vitamin supplement injections to ameliorate intestinal inflammation and protect rats against MTX-induced mucositis. Vitamin C and B2 efficacy has been tested by da Silva Ferreira et al. [214] using an in vitro bacterial growth model, and an in vivo rat model. Both vitamins were able to enhance the growth of gut bacteria, leading to enrichment of Blautia coccoides and Roseburia intestinalis. However, despite the fact that vitamin C ameliorated clinical symptoms of mucositis, neither of the vitamins was able to modulate the course of MTX-induced mucositis, as assessed by citrulline plasma levels [214].

Salecan is a non-toxic water-soluble β-glucan exhibited dose-dependent effects against intestinal mucositis in rats treated with MTX [215]. Salecan treatment inhibited oxidative stress through the effective scavenging of ROS, therefore maintaining mucosal architecture and integrity. Similarly, sodium alginate, a salt of alginic acid found in algae, was reported to protect intestinal architecture, and even mitigate hematologic side effects of MTX treatment in rats [216].

While probiotics have not been extensively explored as preventive tools for MTX-induced multi-organ toxicity, they might have useful properties in preventing intestinal damage. In an MTX toxicity model in rats, cow’s milk yogurt fermented with Lactobacillus johnsonii and sheep’s milk yogurt fermented with a combination of Lactobacillus bulgaricus and Streptococcus thermophilus were able to improve small intestinal barrier function and prevent MTX damage to the small intestine [217].

### 4.4. Pulmonary Toxicity

While the mechanism behind MTX pulmonary toxicity is not well understood, it is likely to be an idiosyncratic reaction and not linked to folate antagonism, as it appears in both high-dose and low-dose treatment schemes [25,218,219]. The presence of mononuclear cell infiltration and inflammatory granulomas in lung injury of animal models upon MTX treatment suggests that MTX pneumonitis represents a hypersensitivity reaction [213,218,219,220]. Pulmonary protective nutraceuticals have not been studied extensively, with few compounds being tested in experimental setting.

Polydatin, also known as picedin, is a major resveratrol derivative found in grape juice. Polydatin’s antioxidant and anti-inflammatory properties increased cellular antioxidant status and reduced inflammation and fibrosis in rats [31]. In rat models of pulmonary oxidative damage, the effects of lutein and alfa lipoic acid elicits antioxidative and anti-inflammatory features in lung tissue, by increasing glutathione levels, and decreasing proinflammatory cytokines [221,222]. 

A synopsis of the main natural components used to counteract multi-organ injury induced by MTX toxicity is provided in Table 3.

While cost-wise and therapeutically effective, nutraceutical therapy for MTX-induced toxicity in humans has not been fully explored in terms of high-dose-induced adverse effects and dose accumulation of multiple nutraceuticals simultaneously administered.

Most studies have tested the effect of the nutraceutical alone on the health of animal models, with no ill effect detected. However, there are not enough studies that explored the effect of nutraceuticals on the pharmacological profile of MTX or other common medications [10]. While most nutraceuticals come from sources that humans have been regularly consuming with no adverse effects, it is unclear if those same compound administered in higher doses are as safe [131]. Thus, before any clinical trials can begin, further testing, with longer periods of nutraceutical administration needs to be performed, in order to assess the effects of long-term administration of nutraceuticals.

## 5. Conclusions and Future Perspectives

MTX is, and will continue to be clinically relevant for years to come due to a wide sera of therapeutic applications, from chemotherapeutical agents for malignant hemopathies, osteosarcoma, breast cancer, etc. [47,57,58], to anti-inflammatory medication for vasculitis, systemic lupus erythematosus, multiple sclerosis, transplantation surgeries [59,60], and further as a disease-modifying agent for RA, JIA, and psoriasis [57,58,61]. However, MTX therapy comes with pitfalls, making it less than perfect. The severe side effects associated with MTX use can occur in both low- and high-dose regiments, often leading to temporary or permanent interruption of MTX medication [47,57,61,128], which limits its clinical potential in patients with urgent need of it [47,61,127,128].

Clinicians could benefit from adjuvant compounds which are designed to reduce the severity and frequency of adverse effects and can be used as a MTX co-treatments. Emerging nutraceutical compounds demonstrated translational relevance as adjuvant therapy into experimental models of MTX toxicity posing several advantages that make them good candidates for such a task [133,134,135,136,137,138,139,140]. With a wide variety of natural compounds that do not pose a health risk for patients, are easy to administer, and relatively inexpensive, clinicians could effectively prevent therapeutic limitations and debilitating side effects of MTX treatment, by using nutraceuticals. Therefore, to take these experimental data from bench to bedside, further clinical trials to explore the clinical potential of such compounds in patients treated with MTX are needed.

## Figures and Tables

**Table 1:** Synopsis of MTX as disease-modifying agent in multiple disease conditions.

Disease	End-Organ Effect	Molecular Mechanism	Efficient Dose Indicated	Most Common Adverse Effects
Acute lymphoblastic leukemia	Neoplastic cells, abnormally fast-dividing cells	DHFR inhibition, disruption of de novo nucleotide biosynthesis and DNA replication, resulting in cell death	Induction 3.3 mg/m^2^ Maintenance 30 mg/m^2^/week	- Liver enzymes elevation - Hepatic fibrosis/cirrhosis - GI bleed - Diarrhea - Ulcerative stomatitis - Leukopenia - Anemia - Aplastic anemia - Pancytopenia - Pneumonia - Pulmonary fibrosis - Renal insufficiency - Hematuria - Toxic epidermal necrolysis - Nausea - Drowsiness
Acute promyelocytic leukemia				
Meningeal leukemia			12 mg/m^2^/every 2–5 days until the cell count of the CSF returns to normal	
Burkitt’s lymphoma and other non-Hodgkin’s lymphomas			10–25 mg/day 4–8 days	
Mycosis Fungoides			2.5–10 mg/day orally or 50 mg/week i.m.	
Epidermoid cancers of the head and neck			30–40 mg/m^2^/week i.v	
Early-stage breast cancer				
Squamous cell carcinoma				
Small cell carcinoma				
Osteosarcoma			12 g/m^2^ i.v.	
Chorioadenoma destruens			15–30 mg/day 5 day course	
Hydatidiform mole				
Severe psoriasis	T cells B cells	Adenosine accumulation, inhibition of T-cell activation, downregulation of B cells	Single dose 10–25 mg/week	
Rheumatoid arthritis			Single dose 7.5 mg/week	
Polyarticular course juvenile rheumatoid arthritis			Single dose 10 mg/m^2^/week	

References [47,57,58,61,62].

**Table 2:** Gut microbiota-related changes following treatment with MTX and the associated epithelial changes.

Gut Microbiota Changes in Subjects Exposed to MTX	Intestinal Epithelial Changes	Samples	Technique	References
↑ *Peptostreptococcaceae and Porphyromonadaceae* ↓ *Ruminococcaceae, Erysipelotrichaceae*	NR	22 Male Sprague Dawley rats 7 to 8 weeks old	Fecal DNA extraction and sequencing	[26]
↓ *Lactobacillus* ↑ *Muribaculaceae*	↓ ZO-1, claudin-1, and E-cadherin	8-week-old male mice (6 per group)	Fecal DNA extraction and sequencing	[32]
↓ *H. filiformis and Bacteroides* sp. ↑ *P. intermedia*	NR	21 RA patients at pre and post-MTX + tripterygium glycosides	Metagenomic shotgun sequencing	[121]
↑ Subdoligranulum ↓ *Rikenellaceae, Veillonellaceae, Bacteroidales_S24-7_group, Alistipes Prevotellaceae_NK3B31_group*	NR	Fecal samples from 29 children with JIA treated with MTX	DNA extraction, amplification, and sequencing	[122]
↓ *Enterobateriales*	NR	11 patients with RA receiving MTX	DNA extraction and metagenomic sequencing	[124]

Abbreviations: JIA, juvenile idiopathic arthritis; MTX, methotrexate; NR, not reported; RA, rheumatoid arthritis; ZO-1, zonula occludens-1.

**Table 3:** End-organ effects of nutraceuticals used in experimental models of MTX-induced toxicity.

Name	Model	Class	Source	Therapeutic Effects	Ref.
Choline	In vivo liver toxicity rat model	Vitamin/nutrient	Multiple sources in meat and plants	↑ PCho, GroPCho, and betaine	[155]
Gossypin/gossypentin	In vivo liver toixicity rat model	Flavonoid/plant extract	Hibiscus sabdariffa	↓BAX, TGF-β, caspase 3, and NF-κB ↓hepatic fibrosis	[161]
Epicatechin/Catechin	In vivo liver toxicity mice model	Flavonoid/plant extract	Mimosa catechu	↓ IL-1β, TNF-α, and NO ↓ MDA GSH level and activity level of catalase, SOD, and GPx ↑	[30]
Thiamine and thiamine pyrophosphate	In vivo liver toixicity rat model	Vitamin	Whole grains, legumes, and some meats and fish	Thiamine no protective effects reported TPP effects on: MDA and MPO ↓ GSH and SOD ↑	[178]
Thymoquinone	In vivo liver toixicity rat model	Plant extract	Nigella sativa	↓TNF-α, NF-κB COX-2 expressions ↓MDA ↑glutathione and catalase	[158]
Ferulic acid	In vivo liver toxicity mice model	Plant extract	Ferula communis	↓MDA, IL-6, and TNF-α ↓accumulation of inflammatory cells ↓nuclear pyknosis ↑GSH, CAT, TAC	[159]
Rhein/cassic acid	In vivo liver toxicity rat model In vitro normal human hepatocyte (L02 cells) model	Plant extract	Rheum undulatum, Rheum palmatum, Cassia reticulata	↑cell survival rate ↓apoptosis ↑Nrf2, Bcl-2, HO-1 and GCLC ↓Bax ↓NF-κB, TNF-α and caspase-3	[160]
Berberine	In vivo liver toixicity rat model	Plant extract	Berberis vulgaris, Berberis aristata, Mahonia aquifolium, Hydrastis canadensis, Xanthorhiza simplicissima, Phellodendron amurense	↓MDA, PC, NO levels and MPO activity ↑GSH level, SOD, GPx and CAT activities	[162]
Resveratrol	In vivo liver toixicity rat model	Plant extract	Skin of grapes, blueberries, raspberries, mulberries, and peanuts	↓MDA levels, MPO and TF activities and collagen contents ↑GSH ↓TNF-α ↓TBARS, CAT, and GST	[152,163]
Ginko biloba	In silico bio computational model In vivo liver toxicity rat model	Plant extract	Ginko biloba tree	↓caspase-3, JNK and TNF-α ↓apoptosis ↑GSH and GST in silico: drug-receptor interactions stabilized by a low energy value and with a good number of hydrogen bonds	[166]

Abbreviations: BAX, BCL2-associated X protein; Bcl-2, B-cell lymphoma-2; caspase-3, cysteine aspartic acid-specific protease 3; CAT, catalase; COX2: cyclooxygenase-2; GCLC, glutamate-cysteine ligase catalytic subunit; GPx, glutathione peroxidase; GroPCho, glycerophosphocholine; GSH, gluthatione; GST, glutathione S-transferase; HO-1, heme oxygenase 1, IL-1β, interleukin 1 beta; IL-6, interleukin-6; JNK, c-Jun N-terminal kinases; MDA, malondialdehyde; MPO, mieloperoxidase; NF-κB, nuclear factor kappa-light-chain-enhancer of activated B cells; NO, nitric oxide; Nrf2, erythroid 2-related factor 2; PCho, phosphocholine; SOD, superoxide dismutase, TAC, total antioxidant capacity; TBARS, thiobarbituric acid reactive substances; TF, tissue factor; TNF-α, tumor necrosis factor-alpha; TNF-β, tumor necrosis factor-beta; TPP, thiamine pyrophosphate.

## Data Availability

Not applicable.
